# Inverted Uterus of Forty Years Standing, Reduced in Half an Hour,—A New Method

**Published:** 1878-05

**Authors:** 


### Inverted Uterus of Forty Years Standing Reduced in Half an Hour. A New Method.

Dr. J. H. Tate reports in the *Cincinnati Lancet and Observer* the following interesting case: *

"Mrs. Bernhard; German; æt. 78; widow; below medium size; physical powers well preserved; health good. History: Came to America some forty years since, and gave birth to her last child in this country when she was thirty-six years of age. At that time she thinks the inversion took place, since which her womb has never been right. About six years ago she had a severe fall, and ever afterward there has been a complete prolapsus of the vagina and womb. Has never consulted any physician in regard to her case, because she was always ashamed to let her condition be known.

Present state: There is complete procidentia uteri, with both vesicocele and rectocele. The whole surface of the vagina down to the os has been converted into common integument. At the lower edge of the vagina there is a superficial ulcer an inch and a

* This case is illustrative of the position long held by Prof. White, that chronic inversion of the uterus may be reduced at any period. Ed.

half in length by half an inch in breadth. At the most dependent part of the vagina hangs the uterus completely inverted, hypertrophied to the size of a duck's egg, feeling quite firm, and its inner surface (now outside), covered and moistened by a glairy, mucosaneous fluid. The urethra now leads from above downwards, and in consequence the urine is passed with much difficulty.

July 29. Patient was put under chloroform, and Dr. Tate proceeded to make the reduction. Present of the Hospital Staff, Drs. Young, Carson and Dandridge, and Dr. Haile of the city. The fore and middle finger of the right hand were introduced into the rectum and carried up far enough to allow them to be placed above the rim of the os uteri, and thus secure a point of counter pressure. The balls of both thumbs were then placed against the fundus, and strong pressure made to push it inward. Working in this way for some time the fundus was found to yield a little, but the fingers in the rectum became fatigued, and the cervex would slip away from them toward the opposite side. At this point Dr. Dandridge suggested that if the finger of the left hand were inserted into the bladder probably the parts could be held more firmly and the counter pressure be made complete. The urethra was dilated in a minute or two, and then the forefinger of the left hand carried through it into the bladder, and placed over the rim of the os, directly opposite where the fingers rested which had been introduced into the rectum. The uterus was thus firmly held between the fingers in the rectum and bladder at the cervical end, and the balls of the thumbs resting over the fundal extremity, and now the pressure on the fundus was renewed. In a few minutes a decided impression was made, the fundus became deeply indented. At this point a star candle with a soft rag wound around its end was planted against the fundus in place of the thumbs, and with strong pressure made against that part the inversion was soon completely reduced.

The whole operation did not occupy more than thirty minutes. To insure its retention a silver suture was made to bring together the lips of the os uteri, and then the prolapsus was reduced. A sponge covered with oil silk smeared with lard was introduced into the vagina to prevent subsequent procidentia; the woman placed in bed and ordered to maintain the recumbent position. If pains ensued opiates were to be given.

August 21.—Patient has been up and about the ward for several days; it is found that the sponge will not retain the vagina in place; the uterus, however, is still in proper position. Recommended to the patient, as a means of permanent relief, the operation of perineorrhaphy.

September 1.—The patient will not consent to any further operative procedures, and left the hospital with no further trouble than the procidentia, the inversion having been completely cured."

